# The Effects of Cold-Plasma Technology on the Quality Properties of Fresh-Cut Produce: A Review

**DOI:** 10.3390/foods14020149

**Published:** 2025-01-07

**Authors:** Yuanpeng Li, Xinmeng Huang, Yuting Yang, Ailikemu Mulati, Jingyang Hong, Jiayi Wang

**Affiliations:** College of Life Science and Technology, Xinjiang University, Urumqi 830046, China; 15983612718@163.com (Y.L.); huangxm0415@163.com (X.H.); 13350321557@139.com (Y.Y.); alkam@stu.xju.edu.cn (A.M.); hongjingyang2017@126.com (J.H.)

**Keywords:** cold plasma, non-thermal processing, fresh-cut fruits and vegetables, retain freshness

## Abstract

With improving economic conditions, consumer demand for fresh-cut produce is rising. The development of the fresh-cut industry has been hindered by pathogenic contamination and quality deterioration. Scientific communities have developed novel preservation technologies for fresh-cut produce. As an innovative non-thermal processing method, cold plasma effectively preserves the nutritional value and inactivates pathogens in fresh-cut produce. This review delineates the principles of cold-plasma generation and concludes with the primary factors influencing its efficacy. These factors include the specifications and parameters of the equipment utilized, the properties of the conductive gas utilized, the method of treatment, and the intrinsic properties of a sample subjected to treatment. Furthermore, this review delineates various scenarios for cold-plasma applications. This review focuses on its effects on enzymatic activities (including peroxidase, polyphenol oxidase, and pectin methylesterase), pathogenic microorganisms, and nutritional value. This review concludes with the potential application of cold-plasma technology in the processing of fresh-cut products. This study proposes advancing plasma technology in fresh-cut produce processing by (1) optimizing cold-plasma parameters for diverse fruit and vegetable varieties and (2) scaling up to facilitate industrial application.

## 1. Introduction

Ready-to-eat fruits and vegetables, also referred to as fresh-cut produce, offer the advantages of convenience and diversity. These characteristics are appealing to numerous contemporary consumers, particularly workers with limited temporal resources. The progressive substitution of conventional processed foods with minimally processed, fresh, and nutritious alternatives is predominantly attributable to heightened consumer awareness regarding food safety. This shift in consumer awareness has consequently facilitated the rapid expansion of the fresh-cut produce [1]. A novel trend in food supply has emerged, emphasizing natural, environmentally sustainable, and nutritious foods. This trend represents a paradigm shift in the conceptualization of food production and consumption [2]. Fresh-cut produce exhibits a higher susceptibility to spoilage than unprocessed raw materials. This heightened perishability is attributable to minimal processing procedures, including peeling, cutting, sanitizing, color preservation, precooling, refrigeration, transportation, and storage. These procedures may potentially increase the risk of contamination by pathogenic microorganisms, including *Salmonella* and *Listeria monocytogenes.* Such contamination may result in diminished nutritional value and pose a substantial health risk to humans. Prolonged exposure to atmospheric environmental conditions may enhance the activity of polyphenol oxidase and ethylene. This phenomenon results in various detrimental effects, including the browning of surfaces and softening of tissues, which consequently reduces the storage longevity of fruits and vegetables [3].

Fresh fruits and vegetables are typically processed utilizing either thermal or non-thermal methodologies. Conventional thermal treatment extends the shelf life of these products by inhibiting respiration and enzymatic activity. By reducing the respiration rate, less energy is consumed by the products, thereby decelerating the aging process. Concurrently, inhibiting enzymatic activity aids in preventing the degradation of essential nutrient substances and maintaining the quality of fresh fruits and vegetables. This approach has been widely utilized in the food industry [4]. However, elevated temperatures can induce irreversible detrimental effects on fruits and vegetables [5]. The decline in the overall quality and consumer appeal of fresh products is attributed to multiple factors, including pathogenic microorganisms, peroxidase (POD), polyphenol oxidase (PPO), pectin methylesterase (PME), color, texture, flavor, and firmness. Non-thermal treatments are predominantly employed to mitigate the deterioration of these products at low or ambient temperatures [6]. In comparison to conventional thermal treatment methods, this approach demonstrates the potential to enhance energy efficiency, improve economic viability, and promote environmental sustainability [7]. Refrigeration at low temperatures was employed to preserve fresh products. However, this methodology may result in cold-induced damage owing to the varying cold sensitivities of different fresh products. Furthermore, researchers have incorporated natural preservatives to enhance the longevity of fresh-cut produce.

In recent years, there has been a substantial increase in awareness regarding safety concerns. This enhancement in safety consciousness, when combined with technological advancements, has played a crucial role in significantly contributing to the development of various novel non-thermal food processing technologies. These innovative technologies encompass a diverse array of options. These technologies include pulsed electric fields, cold plasma, ultraviolet light, ultrasonic waves, and high-pressure processing and related methods. Notably, these techniques exert minimal influence on the organoleptic properties and nutritional composition of food products, while effectively prolonging the shelf life of perishable commodities. Ultraviolet light can be utilized to sanitize food surfaces by effectively inactivating the DNA of pathogenic microorganisms [8]. Ultrasonic waves can be employed for various applications, including the destruction of cellular structures, which results in the inactivation of pathogenic microorganisms and extends the shelf life of fresh-cut produce by cavitation effects [9]. High-pressure processing subjects food to extremely high pressures, inactivating pathogenic microorganisms and extending the shelf life of food products [10].

In this field, cold-plasma technology demonstrates the potential to emerge as one of the more innovative methods in food processing, offering advantages such as safety, environmental sustainability, and the absence of deleterious residues. This technology has demonstrated applications across diverse industries, including the textile sector, where it enhances the functional properties of polymers and improves adhesion [11], and in the medical field, where it is utilized for the surface purification of medical equipment and treatment of waste liquids [12]. Cold-plasma technology is gaining significance in the fresh-cut industry as it prolongs the shelf life of food products by inactivating pathogenic microorganisms and inhibiting the activity of oxidative and respiratory enzymes without nutrient destruction, the degradation of sensory attribute characteristics, tissue softening, and browning [13]. Cold-plasma technology has been utilized to incorporate silver and triclosan into food packaging films to produce antibacterial packaging film materials [14].

Plasma generates ultraviolet radiation, electric fields, and active radical species, thereby demonstrating efficacy in food sterilization [15]. Besides inactivating pathogenic microorganisms, numerous studies have shown that PPO in fresh-cut apple [16] and POD in fresh-cut apples and potatoes [16] were observed to be inactivated by cold plasma. The present review focuses on the effect of cold-plasma technology on the quality of fresh-cut produce. We hope that this technology will contribute to future research endeavors. However, a notable limitation of this review is its omission of considerations regarding the integration of cold-plasma technology with other innovative non-thermal processing techniques (ionizing radiation, UV, and pulsed light) for fresh-cut produce. Furthermore, technology interoperability represents a significant avenue for future research, as it possesses the potential to effectively enhance food safety in fresh-cut produce.

## 2. Cold-Plasma Technology Overview

### 2.1. Definition of Plasma

Plasma is conventionally classified as the fourth state of matter, in addition to solid, liquid, and gas, and is characterized as an “ionized gas”. It exhibits electrical conductivity and responsiveness to electromagnetic fields [17]. When high-voltage electricity interacts with gas molecules, it imparts sufficient energy to remove electrons from the atoms, resulting in a mixture of ions and free electrons [18].

Plasma can be categorized into hot plasma (1000 to 10,000 K) and cold plasma (300 to 500 K). Cold plasma, also termed nonequilibrium plasma, meets the requisite conditions for non-thermal food product processing. This phenomenon is attributed to the fact that the energy generated by the plasma is predominantly concentrated in the electrons produced, rather than being uniformly distributed throughout the ion flow. As a result, deleterious effects on food are minimized [19].

### 2.2. Generation of Cold Plasma

The fundamental principle governing the generation of cold plasma is based on the ionization of the carrier gas. During this complex process, ionization of gaseous molecules occurs. The ionization process is accompanied by ultraviolet emission and the generation of high-energy electrons, ions, and various reactive species [20]. These reactive components can interact with both organic and inorganic substances, thereby facilitating various chemical and biological effects [21]. For instance, air plasma is rich in ozone, hydroxyl groups, oxygen, and nitrogen radicals, which effectively decompose contaminants and thereby exhibit sterilization effects [22].

Various carrier gases demonstrate distinct effects in cold-plasma applications. Researchers generally consider carrier gases to encompass a variety of gases, including argon, atmospheric air, and nitrogen. Notably, the utilization of a mixture of nitrogen and argon at ambient temperature and low pressure has demonstrated significant efficacy. At present, the use of atmospheric air as a carrier gas for cold-plasma generation has proven to be cost-effective and highly productive in food processing applications. Concurrently, the presence of inert gases can effectively mitigate the oxidative browning phenomenon when food is exposed to atmospheric conditions [23].

In recent years, the distinctive advantages of cold-plasma technology have garnered substantial attention in the food processing field [24]. Cold plasma generated at atmospheric pressure has demonstrated increased applicability in food processing and industrial sectors. Cold plasma, characterized by a gas temperature approximating the ambient temperature, is generated through various discharge methods at atmospheric pressure, with the primary generation techniques being dielectric barrier discharge (DBD) and atmospheric-pressure plasma jet. Both methods demonstrate relative maturity in the market [25]. The DBD technique comprises a dielectric material and two electrodes that are structured in a configuration analogous to a capacitor. Common dielectric materials employed in DBD systems include glass, ceramics, various polymers (polypropylene, polyethylene, polyvinyl chloride, and polytetrafluoroethylene), metal oxides (alumina and silicon dioxide), and paper-based materials. The dielectric constants of these materials exhibit variability, which indirectly influences the discharge process within the DBD systems. Notably, materials exhibiting higher dielectric constants tend to generate higher electric field strengths. The presence of a dielectric barrier between the electrodes inhibits corrosion and facilitates the generation of multiple discharges [26]. The DBD generates cold plasma through discharge at atmospheric pressure by utilizing a specialized alternating current (AC). The atmospheric-pressure plasma jet comprises a nested pair of quartz tubes with a central silver electrode and a ring electrode (or ground), which are positioned at a distance of several millimeters from each other. A pressure differential is applied between the two electrodes to facilitate the ionization of helium, hydrogen, or nitrogen [27]. Research has demonstrated that cold plasma exhibits high efficacy in inactivating fungi and pests on fresh fruits and vegetables, eliminating the necessity for additional chemical preservatives, and improving environmental conditions, thereby maximizing the preservation of the food’s original quality [28].

### 2.3. Factors Affecting Cold-Plasma Treatment Effectiveness

An analysis of extant research indicates that the efficacy of cold-plasma treatment is influenced by multiple factors. These factors primarily encompass the specifications and parameters of the utilized equipment (voltage, power, and current), gas mixture effect (gas composition and relative humidity), method of treatment, intrinsic properties of a sample subjected to treatment, and additional relevant factors.

#### 2.3.1. Specifications and Parameters of the Utilized Equipment

**Figure 1:**
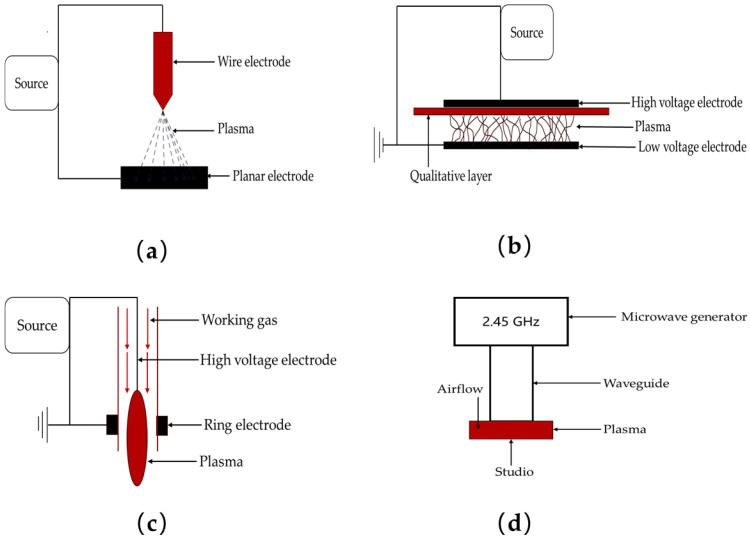
Schematic description of discharge modes for the atmospheric cold plasma. (**a**) Corona discharges; (**b**) dielectric barrier discharge; (**c**) plasma jet; (**d**) microwave plasma sources.

The specifications and parameters of the utilized equipment in the process of cold-plasma generation play a crucial role in determining its efficacy. Various apparatuses with differing specifications can result in substantial variations in the outcomes of cold-plasma generation (Figure 1). Equipment with suboptimal specifications or improperly adjusted parameters may significantly reduce the effectiveness of cold-plasma generation. Conversely, when an optimal combination of specifications and parameters is employed, cold plasma can demonstrate high efficacy in achieving the intended objectives. For example, the type of operating current (DC or AC) is applicable in low-atmospheric-pressure environments; DC is limited to driving arcs and tip coronae under atmospheric-pressure conditions, whereas AC is necessary for equipment susceptible to resistive re-impedance. Hertwig [29] observed that radio frequency (RF) plasma and microwave (MW) plasma were applied to the external surface of black pepper grains. The RF plasma utilized argon as the carrier gas, operating at a frequency of 27.2 MHz, whereas the MW plasma employed air as the carrier gas, functioning at a frequency of 2.8 GHz. The results demonstrated that the sterilization efficacy of the RF plasma was significantly superior to that of the MW plasma. The variations in efficacy can potentially be attributed to the heterogeneous types of cold-plasma generators, diverse carrier gases utilized, and disparate operational frequencies employed. These factors significantly influence the sterilization efficacy of the active components generated during the process.

#### 2.3.2. Gas Mixture Effect

Cold plasma is typically generated in an environment that contains air, nitrogen, or inert gases. The selection of gas composition is a critical factor as it exerts a significant influence on the generation process. Furthermore, the relative humidity of the carrier gas plays a significant role in determining the efficacy of cold-plasma applications. For instance, specific gas mixtures may enhance the reactivity of cold plasma, whereas high relative humidity can increase its stability and efficacy. Patil [30] investigated the inactivation effects of cold plasma generated using various carrier gases on *B. atrophaeus* spores. The findings demonstrated that the number of *B. atrophaeus* spores decreased by 2.5, 1.2, and 6.3 log CFU/cm^2^ after exposure to cold plasma produced by air, a nitrogen–oxygen mixture (9:1), and a nitrogen–oxygen–carbon dioxide mixture (1:6:13), respectively, after one minute of treatment. The observed differences in inactivation rates may be attributed to the diverse active substances generated during the discharge of different carrier gases, which subsequently influence the bactericidal effect.

Patil [30] observed that the lethality of the generated cold plasma against bacteria progressively increased with a gradual increase in the relative humidity of the discharge medium. Specifically, at relative humidity levels of 5%, 15%, and 25%, the application of cold plasma resulted in decreased bacterial activity of 5.2, 6.3, and 6.8 log CFU/cm^2^, respectively, when directly applied to *B. atrophaeus* spores for a duration of one minute. This phenomenon may be attributed to the elevated concentrations of hydroxide ions and ozone ions, which are associated with higher relative humidity levels and enhance the bactericidal efficacy of cold plasma.

#### 2.3.3. Method of Treatment

**Figure 2:**
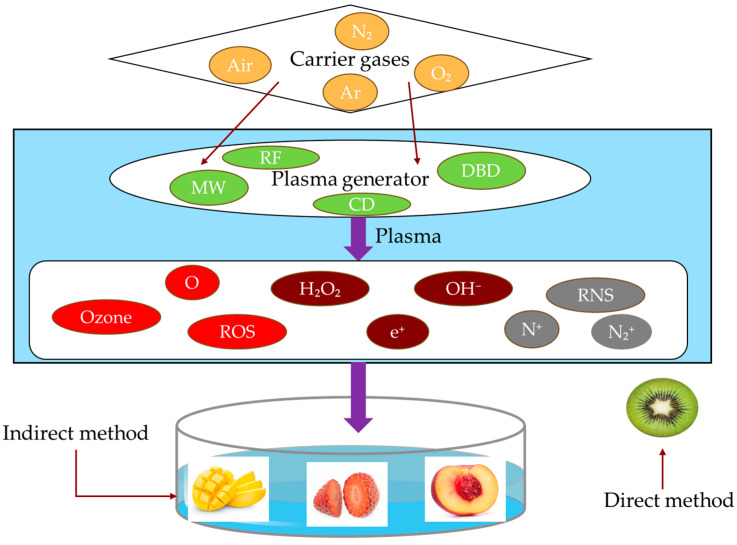
Schematic diagram of cold-plasma generation.

The application of cold-plasma technology for food processing is typically implemented using two primary methods: direct and indirect methods (Figure 2). In the direct approach, food items are exposed to cold-plasma discharge equipment, allowing active radicals and charged particles to come into immediate contact with the exterior of the food. Conversely, the indirect approach involves the introduction of a specific substance, such as water, into the cold-plasma discharge apparatus prior to its application in food. This specific substance becomes saturated with active radicals, after which the food is immersed in a medium to achieve sterilization. Mendes-oliveira [31] observed that following the direct and indirect treatment of foodstuffs with cold-plasma generated by air discharge for a duration of one minute, the activities of *B. subtilis* spores decreased by 2.2 and 0.9 log CFU/cm^2^, respectively. This reduction may be attributed to the dissolution of specific active radicals and other substances generated during indirect treatment of the aqueous solution. As a result, the concentration of these active substances reaching the surface of foodstuffs is insufficient, resulting in inadequate sterilization. Overall, sterilization efficacy was determined to be unsatisfactory.

#### 2.3.4. Intrinsic Properties of a Sample Subjected to Treatment

The intrinsic properties of a sample constitute a significant factor that exerts a notable influence on the outcomes. Multiple aspects of a sample, including its dimensions, morphology, composition, and surface characteristics, can exert a substantial impact. The surface roughness of a sample may potentially influence its susceptibility to the effects of cold plasma. Furthermore, the surface area of a sample may have affected the uniformity of the treatment. Consequently, consideration of the intrinsic properties of a sample is crucial for achieving optimal outcomes when utilizing cold-plasma technology. Pina-perez [32] found that the activity of *B. subtilis* spores exhibited a positive correlation with the thickness of the biopolymer matrix in which the food is located. When *B. subtilis* spores were inoculated onto sterile Petri dishes and corn starch Petri dishes, their activities decreased by 4.2 and 2.4 log CFU/cm^2^, respectively. This reduction may be attributed to an increase in the thickness of the biopolymer layer, which impedes the diffusion rate of charged particles and active radicals, consequently resulting in an inability to achieve the intended sterilization effect.

Kim [33] investigated the positive correlation between the vital activity of *B. cereus* spores and their surface area when subjected to cold-plasma treatment on the surface of red pepper. This phenomenon may be attributed to the reduced surface area of food, which results in higher concentrations of reactive oxygen species and reactive nitrogen species generated per unit area during cold-plasma discharge, thereby enhancing disinfection efficacy. Liu [34] reported that this study examined the influence of moisture activity levels in food on the effectiveness of cold-plasma sterilization. These findings demonstrated that the application of cold-plasma treatment to food surfaces results in the increased production of hydroxide ions, ozone, and peroxide hydroxyl radicals in foods with higher water activity. This results in enhanced sterilization and preservation.

The analysis of the aforementioned four aspects indicates that the specifications and parameters of the utilized equipment, gas mixture effect, method of treatment, and intrinsic properties of a sample subjected to treatment significantly influence the efficacy of the cold-plasma technology. Therefore, in the implementation of cold-plasma technology for food processing applications, it is essential to comprehensively consider these factors. Through a thorough evaluation of these aspects, effective sterilization and preservation of product freshness can be achieved (Table 1). Simultaneously, it can optimize the original nutritional components of food. This method not only ensures food safety and quality but may also address the increasing demand for nutritious and healthful food products.

## 3. Research Status

### 3.1. The Impact of Cold-Plasma Technology on the Enzymatic Activity of Fresh-Cut Fruits and Vegetables

In fresh-cut fruits and vegetables, the enzymatic activities of POD, PPO, and PME are significantly elevated. These enzymes play a crucial role in the degradation process of fresh-cut products. The inactivation of enzymatic activity in these fresh-cut products is directly associated with the application of cold-plasma technology. This process involves electrons, ions, and free radicals, as well as other reactive components. The mechanisms underlying this inactivation involve the modification of amino acid residues within the peptide chains of proteins, the disruption of the enzymes’ tertiary structures, and alterations to the active sites that interact with the reactive substances.

Surowsky [55] demonstrated that the treatment of solutions containing POD and PPO utilizing plasma jet-generated cold plasma. As a result, the α-helix content of POD and PPO decreased by 29.5% and 19.1%, respectively. Furthermore, hydroxylation of tryptophan residues was observed due to the action of the active substances. This phenomenon may be attributed to the reduction or loss of the enzymatic activity of POD and PPO as a consequence of cold-plasma treatment. Tappi [16] demonstrated that the browning area of kiwifruit subjected to plasma treatment was reduced by 60%, and enzyme activity decreased by 58% after 4 h of storage under conditions identical to those of the control group. Furthermore, the application of cold plasma to the surfaces of fresh melons for 30 min resulted in 18% and 5% reductions in POD and PME activities, respectively, compared to the control group. Tappi [16] observed that the PPO activity of the first two apple varieties, Pink Lady and Red Fuji, was significantly reduced following a 30 min treatment with cold plasma. A control group without treatment was used for comparison. Experimental measurements indicated a substantial decrease in PPO activities for the Pink Lady and Red Fuji apples; conversely, the PPO activity of the freshly cut Snake Fruit demonstrated an increasing trend.

Bussler [35] reported that the application of cold-plasma treatment to solutions containing POD in fresh apples and potatoes effectively inhibited tissue browning and minimized nutrient loss. The PPO activity in apples and potatoes was reduced by 65% and 80%, respectively. Furthermore, the more structurally stable POD exhibited reductions of 66% and 85% in apples and potatoes, respectively, following cold-plasma treatment for durations exceeding 10 min.

### 3.2. The Impact of Cold-Plasma Technology on Pathogenic Microorganisms Present on the Surfaces of Fresh-Cut Fruits and Vegetables

Fresh-cut fruits and vegetables are susceptible to contamination by pathogenic microorganisms, including *B. cereus*, *E. coli* O157:H7, *Shigella*, and *Y. enterocolitica*, which can result in the deterioration of product quality [56].

Cold plasma comprises a range of biologically active substances, such as ozone, reactive oxygen species, free radicals, and nitrogen dioxide. These active substances undergo physical and chemical reactions with microbial cell membranes, resulting in the disruption of the cell membrane structure. Concurrently, alterations in osmotic pressure both intracellularly and extracellularly facilitate the penetration of these active substances into the cell. This process elicits an oxidative stress response within the cell, ultimately resulting in the mortality of pathogenic microorganisms. This method constitutes the primary approach for the inactivation of pathogenic microorganisms at ambient temperature [57]. Furthermore, research has identified three hypothesized mechanisms through which cold plasma induces pathogenic microorganism inactivation: the interaction between microorganisms and oxygen free radicals results in the formation of alkaline complexes, which subsequently induce oxidative denaturation of cellular DNA [58]; unsaturated fatty acids exhibit structural instability and are susceptible to destruction by hydroxyl radicals, which can result in lipid peroxidation. Additionally, amino acids undergo oxidation reactions, resulting in protein denaturation and alterations of protein tertiary structure [59].

Highly reactive free radicals, such as reactive nitrogen and reactive oxygen species, can undergo intense chemical interactions with cell membranes. These interactions cause damage that can ultimately result in cellular death. This phenomenon, referred to as etching, is influenced by the properties of the charged particles and the reactants involved. For example, charged particles in Gram-negative bacteria accumulate on the surface of the cell membrane, resulting in their mutual repulsion. As the repulsive force increases, the mechanical strength of the cell membrane decreases, ultimately resulting in rupture due to the electro-osmotic effect [60]. However, Laroussi [61] showed that conducting identical experiments with Gram-positive bacteria, as opposed to Gram-negative bacteria, resulted in no significant alterations in the Gram-positive strains. This phenomenon can be attributed to the cellular structural features of Gram-positive bacteria, which have thicker cell walls and contain a larger amount of peptidoglycan. These features facilitate extensive cross-linking, resulting in a robust mesh-like structure.

Sudarsan [36] found that the application of cold plasma generated in the atmosphere enhanced the inactivation of *Salmonella serotypes* and *Escherichia coli* on fresh spinach. Ziuzina [54] observed that *Salmonella typhimurium*, *Escherichia coli*, *Listeria monocytogenes*, molds, and yeasts were rendered undetectable after 10, 60, 100, and 120 s, respectively, following the treatment of tomatoes with cold plasma generated using atmospheric air as the carrier gas. Conversely, aerobic bacterial populations require 300 s to reach undetectable levels. Bhide [37] observed that the populations of *Escherichia coli*, *Salmonella*, and *Enterobacteriaceae* in apple pulp and pericarp were reduced by 80% following a 3 s treatment with cold plasma generated through atmospheric ionization. Erickson [38] subjected tomatoes and strawberries infected with *Listeria monocytogenes* to atmospheric cold-plasma therapy for 2 min. The results indicated that the bacterial population in the tomatoes and strawberries was significantly reduced.

### 3.3. The Impact of Cold-Plasma Technology on the Quality of Fresh-Cut Fruits and Vegetables

Fresh-cut fruits and vegetables contain diverse bioactive compounds, including total phenols, total flavonoids, anthocyanins, and tea polyphenols. These components exhibit antioxidant and anti-aging properties, as well as the potential to prevent cardiovascular diseases. As a result, they constitute an essential component of the diet and significantly contribute to human health.

Cold plasma generates biologically active substances that can induce oxidation and nitrosation reactions in fresh-cut fruits and vegetables, potentially leading to the degradation of their organizational structure and chemical composition as well as affecting their respiration rate and photosynthesis. For instance, anthocyanins, lycopene, carotenoids, and other pigments in fresh-cut fruits and vegetables undergo oxidation, resulting in the darkening of the color of fresh-cut products. Furthermore, due to the cold-plasma-induced oxidative stress on the cells of fruits and vegetables, the respiration rate is enhanced, leading to an accelerated reduction in antioxidant activity [62].

#### 3.3.1. Color and Firmness

Ramazzina [44] demonstrated that in comparison to the control group, the PAW generated by corona discharge plasma source treatment significantly enhanced color retention during the storage of fresh-cut kiwifruit, inhibited the development of dark-zone browning, and did not result in any softening of the texture. Furthermore, no significant difference in antioxidant activity was observed between the two groups. Zhou [53] reported that the application of cold-plasma treatment on fresh-cut fruits and vegetables resulted in a significant delay in texture degradation when compared to the control group. This phenomenon may be attributed to the inhibitory effects of cold plasma on the physiological activity of cell wall hydrolases, which consequently reduces the synthesis of cellulose, hemicellulose, lignin, pectin, and other relevant compounds in fruits and vegetables, thereby enhancing their flavor.

#### 3.3.2. Antioxidant Activity

Matan [45] indicated that the application of cold-plasma treatment to fresh-cut dragon fruits effectively inhibited the proliferation of surface pathogenic bacteria. Furthermore, the synergistic effect of cold plasma and tea polyphenols was found to enhance the total phenolic, protein, and crude fiber content of the fruits. This treatment resulted in a substantial accumulation of gallic acid, protocatechuic acid, and other phenolic compounds, which significantly improved the antioxidant activity and overall quality of the dragon fruits. Chen [63] posited that cold plasma might disrupt cell membranes, thereby facilitating the release of phenolic compounds. Zielinska [46] observed a significant reduction in the total phenolic content of autumn konjac pods as the duration of cold-plasma treatment increased. Concurrently, Khirunenko [64] observed that tomatoes subjected to cold-plasma pretreatment exhibited an increase in total phenolic compounds. This phenomenon may be attributed to the enhanced activity of phenylalanine deaminase following cold-plasma treatment, which facilitates the conversion of phenylalanine to coumaric acid. Subsequently, coumaric acid is further synthesized into a variety of polyphenols through the action of phenylacetone synthetase and hydroxycoumaric acid benzyltransferase [65]. Furthermore, the flavonoid biosynthesis pathway is closely associated with the synthesis of phenolic acids, using phenylalanine as the primary substrate. The application of cold-plasma induction significantly enhances the activities of 4-coumarate-CoA ligase and chalcone synthetase, thereby promoting the production of polyphenols during the storage period of fresh-cut apples [65].

#### 3.3.3. Vitamins

Fresh-cut produce is abundant in vitamins (vitamin A, vitamin C, vitamin B, and thiamine), and vitamins are a significant indicator for measuring the nutritional value of fresh-cut produce [66]. Peeling and cutting may enhance the production of ethylene, which subsequently accelerates respiration and senescence, resulting in the accelerated loss of vitamins. Among all the vitamins, vitamin C exhibits comparatively high susceptibility to processing [67], as it is an antioxidant and prone to redox reactions when subjected to processing operations [68].

Ramazzina [69] demonstrated no significant difference in vitamin C concentration after DBD cold-plasma treatment of fresh-cut kiwifruit. However, a reduction of 7% in vitamin C was found after 4 days of storage of fresh-cut kiwifruit. Wang [48] found that cold plasma (500 V for 0.5 to 4 s) decreased the content of vitamin C in fresh-cut produce. The content of vitamin C in carrot, cucumber, and pear after the treatment of cold plasma was decreased by 3.2%, 3.6%, and 2.8%, respectively. This phenomenon can be attributed to the reaction of vitamin C with ozone, leading to degradation. Khoshkalam Pour [70] researched the effects of DBD plasma treatment on banana at 4.8 to 6.9 kV for 35 to 155 s. The results showed that there was an increase in the concentration of vitamin B6 by cold-plasma treatment. The control samples exhibited a concentration of 0.6 mg/100 g, while the treated samples had a concentration of 0.8 mg/100 g. This phenomenon may be explained by cell wall degradation, which could lead to increased concentrations of vitamin B6.

## 4. Future Prospects and Imperative

The increasing demand for fresh-cut fruits and vegetables is a significant trend that warrants attention. This growing demand highlights the substantial potential of cold-plasma technology. This technology presents innovative methods that play a pivotal role in mitigating food waste and enhancing resource sustainability. These research endeavors, which aim to extend the shelf life of fresh-cut fruits and vegetables, are of considerable academic interest and hold substantial potential for the future of the food industry [71]. The shelf life of food items can be significantly extended through the application of cold-plasma technology, which effectively eliminates pathogenic microorganisms from the superficial layers of food products. This approach holds considerable significance as it not only substantially reduces economic losses that may occur due to spoilage of food items but also contributes to enhancing the safety and quality of food products [57].

## 5. Limitations and Challenges

Notwithstanding the substantial potential of this technology, numerous challenges and limitations persist and necessitate careful consideration. To establish a theoretical foundation for continuous production, it is essential to optimize cold-plasma equipment. This necessitates the consideration of multiple variables, including the morphology, surface characteristics, and nutritional constituents of fresh-cut produce, as well as the characteristics of the carrier gas, treatment methods, and the comprehensive optimization of system mechanisms. Furthermore, the transition of cold-plasma technology from experimental settings to practical commercial applications faces significant challenges. However, in the food processing domain, certain fresh fruits and vegetables require extended exposure to cold plasma to achieve aseptic conditions. However, this extended exposure may result in a deterioration in the quality of fresh-cut produce, potentially impeding the widespread extension of this technology. For instance, it may lead to alterations in texture, color, and flavor. Consequently, it is imperative to develop a new generation of cold plasma or integrate it with other innovative non-thermal food processing technologies to mitigate such adverse effects [62]. Consequently, novel cold-plasma technologies necessitate an innovative design process and rigorous engineering experimentation. Moreover, this advanced technology has another important objective. By elucidating the alterations that manifest in diverse fresh-cut products subsequent to cold-plasma treatment, the efficacy and applicability of cold-plasma technology can be more precisely ascertained to ensure that the technology not only extends shelf life but also maintains food quality and safety.

## 6. The Effect of Cold Plasma on Sensory Attributes and Consumer Acceptability

Fresh-cut produce has gained significant popularity among consumers, with color, firmness, and nutritional value being crucial factors for marketability [72]. This trend calls for non-thermal processing technologies that result in minimal changes due to physical and chemical reactions that affect the “fresh-like quality” of fresh-cut produce [73]. However, the indicators of this quality have only been investigated at the laboratory scale. For example, Trivedi [50] investigated the effects of weight loss, color, texture, and sugar from cold plasma generated using DBD on bananas. The findings demonstrated that the control and cold-plasma-treated bananas showed no significant differences in weight loss, color, texture, and sugar. In a similar study, Lee [51] investigated the color and sensory evaluation effects of cold plasma generated using CD on cherry tomatoes. They found that the control and cold-plasma-treated cherry tomatoes showed no significant difference in color, flavor, and taste. Color is considered a highly influential indicator of consumer acceptability of food’s healthiness and purchase intention [74]. The absence of significant differences in color would indicate minimal effects of cold plasma on fresh-cut produce and may hence be potentially favored by the majority of consumers.

## 7. Conclusions

An increasing variety of fresh-cut fruits and vegetables is being introduced to the market, and cold-plasma technology presents a novel approach to their preservation, addressing the challenges of fresh-cut fruit and vegetable spoilage and resource wastage. This technology demonstrates significant advantages, including extended shelf life, inhibition of pathogenic microorganism growth, and enhancement in food product nutrient content, among other benefits. While practical applications of cold-plasma treatment for fresh-cut fruits and vegetables are not widely commercially available, research on this technology remains of significant importance. Notwithstanding the significant advancements in this technology within laboratory applications and the extant literature, challenges persist in scaling up the technology and enhancing its economic viability. Further research into cold-plasma technology is imperative for addressing the current challenges and establishing its proven applicability in the future.

The analysis and optimization of factors influencing the efficacy of cold plasma can significantly enhance its effectiveness. Concurrently, it should be optimized for cost-effectiveness and align with contemporary technological capabilities. The future application of cold-plasma technology to the preservation of fresh-cut fruits and vegetables is highly probable. Realizing this aspiration will necessitate close collaboration between experimental researchers and commercial developers. However, this process is likely to be prolonged and complex. As scientific and technological advancements progress and economic conditions improve, cold-plasma technology is likely to emerge as the preferred method in the domain of non-thermal food processing.

## Figures and Tables

**Table 1:** The influence of different cold-plasma systems on the properties of fresh-cut produce.

Fresh-Cut Produce	Plasma Source	Feed Gas	Indicators	Results	References
Fresh melon	DBD	Air	POD and PME activities	The POD and PME activities in melon were decreased by 18% and 5%, respectively	[16]
Different fresh apples	DBD	Air	PPO activity	Pink Lady and Red Fuji apples decreased PPO activity; Snake Fruit increased PPO activity	[16]
Fresh apple and potato	MW	Air	PPO and POD activities	The PPO activity in apple and potato was decreased by 80% and 50%, respectively; the POD activity was decreased	[35]
Fresh spinach	DBD	Air	*Salmonella serotypes* and *Escherichia coli*	Significant reduction in bacterial activity	[36]
Tomato	DBD	Air	*Salmonella typhimurium*, *Escherichia coli*, *Listeria monocytogenes*, molds, and yeasts	Bacterial undetectable after 10, 60, 100, and 120 s	[35]
Apple pulp and pericarp	DBD	Air	*Escherichia coli*, *Salmonella*, and *Enterobacteriaceae*	80% reduction in bacterial counts	[37]
Tomato and strawberry	DBD	Air	*Listeria monocytogenes*	The bacterial population in tomato and strawberry were significantly reduced	[38]
Golden Delicious apple	DBD	Air	*Salmonella* and *Escherichia coli*	5.5 and 5.3 log CFU/cm^2^ reduction	[39]
Cantaloupe	DBD	Aerosolized H_2_O_2_	*S. typhimurium*	2.48 log CFU/cm^2^ reduction	[40]
Fresh-cut strawberry	DBD	Air	Total aerobic bacterial count (TABC)	3.98 log CFU/cm^2^ reduction	[41]
Grape tomato	DBD	Aerosolized H_2_O_2_	*S. Typhimurium*	*S. Typhimurium* decreased below detection limit after 8 s of treatment	[41]
Grape	DBD	Air	Fungi (2.07 log CFU/cm^2^)	1.37 log CFU/cm^2^ reduction	[42]
Cherry tomato	DBD	Air	*Salmonella* and *Escherichia coli*	1 and 2 log CFU/cm^2^ reduction	[43]
Fresh-cut kiwifruit	DBD	Air	Physical and chemical properties	Enhanced color retention, inhibition of browning, no softening of texture, and no difference in antioxidant activity	[44]
Fresh-cut dragon fruit	DBD	Air	Antioxidant activity	Increased total phenolic, protein, and crude fiber content	[45]
Autumn konjac pods	DBD	Air	Antioxidant activity	Reduced total phenolic content	[46]
Fresh-cut strawberry	DBD	Air	Antioxidant activity	Antioxidant activity in strawberry was increased by 17.85%	[41]
Blueberry	Plasma jet	Air	Firmness	Reduced firmness after 60 s	[47]
Fresh pear	Plasma jet	Air	Vitamin C	2.8% reduction	[48]
Blueberry	DBD	Air	Sugar, vitamin C, and anthocyanin	Sugar, vitamin C, and anthocyanin in blueberry were increased by 1.5-, 1.5-, 2.2-fold, respectively	[49]
Strawberry	DBD	Air	Vitamin C	Vitamin C in strawberry significantly decreased	[23]
Banana	DBD	Air	Weight loss, color, texture, and sugar	No significant differences in weight loss, color change, texture change, and sugar	[50]
Cherry tomato	CD	Air	Color and sensory evaluation	No significant differences in color, flavor, and taste	[51]
Fresh-cut radish sprout	MW	Nitrogen	Moisture	Moisture in radish sprout significantly decreased	[52]
Blueberry	DBD	Air	Firmness, vitamin C, anthocyanin, and antioxidant activity	Firmness, vitamin C, and anthocyanin in blueberry decreased; antioxidant activity increased	[53]
Strawberry	DBD	Air	Color, pH, TSS, and firmness	No significant differences in color, pH, TSS, and firmness	[54]

DBD: dielectric barrier discharge, MW: microwave plasma sources, CD: corona discharges.

## Data Availability

No new data were created or analyzed in this study. Data sharing is not applicable to this article.

## References

[1] Curutchet A., Dellacassa E., Ringuelet J.A., Chaves A.R., Vina S.Z. (2014). Nutritional and sensory quality during refrigerated storage of fresh-cut mints. Food Chem..

[2] Pinela J., Ferreira I. (2017). Nonthermal physical technologies to decontaminate and extend the shelf-life of fruits and vegetables: Trends aiming at quality and safety. Crit. Rev. Food Sci..

[3] Abadias M., Alegre I., Oliveira M., Altisent R., Vinas I. (2012). Growth potential of *Escherichia coli* O157:H7 on fresh-cut fruits (melon and pineapple) and vegetables (carrot and escarole) stored under different conditions. Food Control.

[4] Coelho M.C.C., Ghalamara S., Campos D., Ribeiro T.B., Pereira R., Rodrigues A.S., Teixeira J.A., Pintado M. (2023). Tomato Processing By-Products Valorisation through Ohmic Heating Approach. Foods.

[5] Xin J., Wang X., Li N., Liu L., Lian Y., Wang M., Zhao R.-S. (2020). Recent applications of covalent organic frameworks and their multifunctional composites for food contaminant analysis. Food Chem..

[6] Picart-Palmade L., Cunault C., Chevalier-Lucia D., Belleville M.-P., Marchesseau S. (2019). Potentialities and Limits of Some Non-thermal Technologies to Improve Sustainability of Food Processing. Front Nutr..

[7] Rifna E.J., Misra N.N., Dwivedi M. (2023). Recent advances in extraction technologies for recovery of bioactive compounds derived from fruit and vegetable waste peels: A review. Crit. Rev. Food Sci..

[8] Gürsu H. (2024). Reusable Smart Lids for Improving Food Safety at Household Level with Programmable UV-C Technology. Sustainability.

[9] Zhang C., Xie J. (2022). Ultrasound-Assisted Slightly Acidic Electrolyzed Water in Aquatic Product Sterilization: A Review. Foods.

[10] Drosou C., Sklirakis I., Polyzou E., Yakoumis I., Boukouvalas C.J., Krokida M. (2024). Processing Fresh-Cut Potatoes Using Non-Thermal Technologies and Edible Coatings. Appl. Sci..

[11] Gupta R.K., Guha P., Srivastav P.P. (2022). Natural polymers in bio-degradable/edible film: A review on environmental concerns, cold plasma technology and nanotechnology application on food packaging—A recent trends. Food Chem. Adv..

[12] von Keudell A., Schulz-von der Gathen V. (2017). Foundations of low-temperature plasma physics—An introduction. Plasma Sources Sci. Technol..

[13] Fan X., Wang W. (2021). Quality of fresh and fresh-cut produce impacted by nonthermal physical technologies intended to enhance microbial safety. Crit. Rev. Food Sci. Nutr..

[14] Pankaj S.K., Bueno-Ferrer C., Misra N.N., Milosavljevic V., O’Donnell C.P., Bourke P., Keener K.M., Cullen P.J. (2014). Applications of cold plasma technology in food packaging. Trends Food Sci. Technol..

[15] Birania S., Attkan A.K., Kumar S., Kumar N., Singh V.K. (2022). Cold plasma in food processing and preservation: A review. J. Food Process Eng..

[16] Tappi S., Gozzi G., Vannini L., Berardinelli A., Romani S., Ragni L., Rocculi P. (2016). Cold plasma treatment for fresh-cut melon stabilization. Innov. Food Sci. Emerg. Technol..

[17] Rahman M., Hasan M.S., Islam R., Rana R., Sayem A., Sad M.A.A., Matin A., Raposo A., Zandonadi R.P., Han H. (2022). Plasma-Activated Water for Food Safety and Quality: A Review of Recent Developments. Int. J. Environ. Res. Public Health.

[18] Niemira B.A. (2012). Cold Plasma Decontamination of Foods. Annu. Rev. Food Sci. Technol..

[19] Stoica M., Sorin C., Visan R., Dreve A. (2024). AI, BlazePod Sensors, and Head Vests Implemented in Assessments on Reaction Time and Gaze Training Program in U10 Football Game. Appl. Sci..

[20] Ravash N., Hesari J., Feizollahi E., Dhaliwal H.K., Roopesh M.S. (2024). Valorization of Cold Plasma Technologies for Eliminating Biological and Chemical Food Hazards. Food Eng. Rev..

[21] Dharini M., Jaspi S., Mahendran R. (2023). Cold plasma reactive species: Generation, properties, and interaction with food biomolecules. Food Chem..

[22] Umair M., Jabbar S., Ayub Z., Aadil R.M., Abid M., Zhang J., Zhao L. (2022). Recent Advances in Plasma Technology: Influence of Atmospheric Cold Plasma on Spore Inactivation. Food Rev. Int..

[23] Misra N.N., Pankaj S.K., Frias J.M., Keener K.M., Cullen P.J. (2015). The effects of nonthermal plasma on chemical quality of strawberries. Postharvest Biol. Technol..

[24] Dufay M., Jimenez M., Degoutin S. (2020). Effect of Cold Plasma Treatment on Electrospun Nanofibers Properties: A Review. ACS Appl. Bio Mater..

[25] Yahaya A.G., Okuyama T., Kristof J., Blajan M.G., Shimizu K. (2021). Direct and Indirect Bactericidal Effects of Cold Atmospheric-Pressure Microplasma and Plasma Jet. Molecules.

[26] Engeling K.W. (2019). Micro-Plasma in Porous Media and Dielectric Barrier Discharges with Advanced Agricultural Applications. Ph.D. Thesis.

[27] Thomas J., Volkov A.G. (2024). Electrochemical Reactions at the Boundary Areas Between Cold Atmospheric Pressure Plasma, Air, and Water. Plasma.

[28] Cheng J.H., He L., Sun D.W., Pan Y., Ma J. (2023). Inhibition of cell wall pectin metabolism by plasma activated water (PAW) to maintain firmness and quality of postharvest blueberry. Plant Physiol. Biochem..

[29] Hertwig C., Reineke K., Ehlbeck J., Knorr D., Schlueter O. (2015). Decontamination of whole black pepper using different cold atmospheric pressure plasma applications. Food Control.

[30] Patil S., Moiseev T., Misra N.N., Cullen P.J., Mosnier J.P., Keener K.M., Bourke P. (2014). Influence of high voltage atmospheric cold plasma process parameters and role of relative humidity on inactivation of *Bacillus atrophaeus* spores inside a sealed package. J. Hosp. Infect..

[31] Mendes-Oliveira G., Jensen J.L., Keener K.M., Campanella O.H. (2019). Modeling the inactivation of Bacillus subtilis spores during cold plasma sterilization. Innov. Food Sci. Emerg..

[32] Pina-Perez M.C., Martinet D., Palacios-Gorba C., Ellert C., Beyrer M. (2020). Low-energy short-term cold atmospheric plasma: Controlling the inactivation efficacy of bacterial spores in powders. Food Res. Int..

[33] Kim J.E., Choi H.-S., Lee D.-U., Min S.C. (2017). Effects of processing parameters on the inactivation of Bacillus cereus spores on red pepper (*Capsicum annum* L.) flakes by microwave-combined cold plasma treatment. Int. J. Food Microbiol..

[34] Liu Y., Sun Y., Wang Y., Zhao Y., Duan M., Wang H., Dai R., Liu Y., Li X., Jia F. (2023). Inactivation mechanisms of atmospheric pressure plasma jet on Bacillus cereus spores and its application on low-water activity foods. Food Res. Int..

[35] Bussler S., Ehlbeck J., Schlueter O.K. (2017). Pre-drying treatment of plant related tissues using plasma processed air: Impact on enzyme activity and quality attributes of cut apple and potato. Innov. Food Sci Emerg..

[36] Sudarsan A., Vithya V. (2024). Unraveling the Determinants of Maternal Well-Being Among Tribal Populations of Kerala: A Systematic Review. J. Racial Ethn. Health Disparities.

[37] Bhide S., Salvi D., Schaffner D.W., Karwe M.V. (2017). Effect of Surface Roughness in Model and Fresh Fruit Systems on Microbial Inactivation Efficacy of Cold Atmospheric Pressure Plasma. J. Food Prot..

[38] Erickson M.C., Liao J.-Y., Webb C.C., Habteselassie M.Y., Cannon J.L. (2018). Inactivation of *Escherichia coli* O157:H7 and Salmonella deposited on gloves in a liquid state and subjected to drying conditions. Int. J. Food Microbiol..

[39] Kilonzo-Nthenge A., Liu S., Yannam S., Patras A. (2018). Atmospheric Cold Plasma Inactivation of *Salmonella* and *Escherichia coli* on the Surface of Golden Delicious Apples. Front. Nutr..

[40] Song Y., Fan X. (2020). Cold plasma enhances the efficacy of aerosolized hydrogen peroxide in reducing populations of Salmonella Typhimurium and Listeria innocua on grape tomatoes, apples, cantaloupe and romaine lettuce. Food Microbiol..

[41] Li M., Li X., Han C., Ji N., Jin P., Zheng Y. (2019). Physiological and Metabolomic Analysis of Cold Plasma Treated Fresh-Cut Strawberries. J. Agric. Food Chem..

[42] Moon A.Y., Noh S., Moon S.Y., You S. (2016). Feasibility study of atmospheric-pressure plasma treated air gas package for grape’s shelf-life improvement. Curr. Appl. Phys..

[43] Timmons C., Pai K., Jacob J., Zhang G., Ma L.M. (2018). Inactivation of *Salmonella enterica*, Shiga toxin-producing *Escherichia coli*, and *Listeria monocytogenes* by a novel surface discharge cold plasma design. Food Control.

[44] Ramazzina I., Lolli V., Lacey K., Tappi S., Rocculi P., Rinaldi M. (2022). Fresh-Cut Eruca Sativa Treated with Plasma Activated Water (PAW): Evaluation of Antioxidant Capacity, Polyphenolic Profile and Redox Status in Caco2 Cells. Nutrients.

[45] Matan N., Puangjinda K., Phothisuwan S., Nisoa M. (2015). Combined antibacterial activity of green tea extract with atmospheric radio-frequency plasma against pathogens on fresh-cut dragon fruit. Food Control.

[46] Zielinska S., Staniszewska I., Liu Z.-L., Zielinska D., Pan Z., Xiao H.-W., Zielinska M. (2023). Effect of cold atmospheric pressure plasma pretreatment on the drying kinetics, physicochemical properties and selected bioactive compounds of okra pods subjected to hot air impingement drying. Dry. Technol..

[47] Lacombe A., Niemira B.A., Gurtler J.B., Fan X., Sites J., Boyd G., Chen H. (2015). Atmospheric cold plasma inactivation of aerobic microorganisms on blueberries and effects on quality attributes. Food Microbiol..

[48] Wang R.X., Nian W.F., Wu H.Y., Feng H.Q., Zhang K., Zhang J., Zhu W.D., Becker K.H., Fang J. (2012). Atmospheric-pressure cold plasma treatment of contaminated fresh fruit and vegetable slices: Inactivation and physiochemical properties evaluation. Eur. Phys. J. D.

[49] Dong X.Y., Yang Y.L. (2019). A Novel Approach to Enhance Blueberry Quality During Storage Using Cold Plasma at Atmospheric Air Pressure. Food Bioprocess Technol..

[50] Trivedi M., Patel K., Itokazu H., Huynh N.A., Jasreen S. (2019). Enhancing shelf life of bananas by using atmospheric pressure pulsed cold plasma treatment of the storage atmosphere. Plasma Med..

[51] Lee T., Puligundla P., Mok C. (2018). Intermittent corona discharge plasma jet for improving tomato quality. J. Food Eng..

[52] Oh Y.J., Song A.Y., Min S.C. (2017). Inhibition of Salmonella typhimurium on radish sprouts using nitrogen-cold plasma. Int. J. Food Microbiol..

[53] Zhou D., Wang Z., Tu S., Chen S., Peng J., Tu K. (2019). Effects of cold plasma, UV-C or aqueous ozone treatment on Botrytis cinerea and their potential application in preserving blueberry. J. Appl. Microbiol..

[54] Ziuzina D., Misra N.N., Han L., Cullen P.J., Moiseev T., Mosnier J.P., Keener K., Gaston E., Vilaro I., Bourke P. (2020). Investigation of a large gap cold plasma reactor for continuous in-package decontamination of fresh strawberries and spinach. Innov. Food Sci. Emerg. Technol..

[55] Surowsky B., Fischer A., Schlueter O., Knorr D. (2013). Cold plasma effects on enzyme activity in a model food system. Innov. Food Sci. Emerg..

[56] Kambhampati Vivek K.V., Singh S.S., Ritesh W., Soberly M., Baby Z., Baite H., Mishra S., Pradhan R.C. (2019). A review on postharvest management and advances in the minimal processing of fresh-cut fruits and vegetables. J. Microbiol. Biotechnol. Food Sci..

[57] Nwabor O.F., Onyeaka H., Miri T., Obileke K., Anumudu C., Hart A. (2022). A Cold Plasma Technology for Ensuring the Microbiological Safety and Quality of Foods. Food Eng. Rev..

[58] Cheng J.-H., Lv X., Pan Y., Sun D.-W. (2020). Foodborne bacterial stress responses to exogenous reactive oxygen species (ROS) induced by cold plasma treatments. Trends Food Sci. Technol..

[59] Dar A.H., Bashir O., Khan S., Wahid A., Makroo H.A. (2020). Fresh-cut products: Processing operations and equipments. Fresh-Cut Fruits and Vegetables.

[60] Mendis D.A., Horanyi M. (2013). Plasma processes at comet Churyumov-Gerasimenko: Expectations for Rosetta. J. Plasma Phys..

[61] Laroussi M., Mendis D.A., Rosenberg M. (2003). Plasma interaction with microbes. New J. Phys..

[62] Barbhuiya R.I., Singha P., Singh S.K. (2021). A comprehensive review on impact of non-thermal processing on the structural changes of food components. Food Res. Int..

[63] Chen Y., Zhang Y., Jiang L., Chen G., Yu J., Li S., Chen Y. (2020). Moisture molecule migration and quality changes of fresh wet noodles dehydrated by cold plasma treatment. Food Chem..

[64] Khirunenko L., Sosnin M., Duvanskii A., Abrosimov N. (2024). Temperature-Induced Transformation of the Atomic Configuration of the BO Defect in Boron-Doped Czochralski Si. Phys. Status Solidi A.

[65] Farias T.R.B., Rodrigues S., Fernandes F.A.N. (2020). Effect of dielectric barrier discharge plasma excitation frequency on the enzymatic activity, antioxidant capacity and phenolic content of apple cubes and apple juice. Food Res. Int..

[66] Li L., Pegg R.B., Eitenmiller R.R., Chun J.Y., Kerrihard A. (2017). Selected nutrient analyses of fresh, fresh-stored, and frozen fruits and vegetables. J. Food Compos. Anal..

[67] Gutierrez Gossweiler A., Martinez-Mier E.A. (2020). Chapter 6: Vitamins and Oral Health. The Impact of Nutrition and Diet on Oral Health.

[68] Chen C., Liu C., Jiang A., Guan Q., Hu W. (2019). The Effects of Cold Plasma-Activated Water Treatment on the Microbial Growth and Antioxidant Properties of Fresh-Cut Pears. Food Bioprocess Technol..

[69] Ramazzina I., Berardinelli A., Rizzi F., Tappi S., Ragni L., Sacchetti G., Rocculi P. (2015). Effect of cold plasma treatment on physico-chemical parameters and antioxidant activity of minimally processed kiwifruit. Postharvest Biol. Technol..

[70] Pour A.K., Khorram S., Ehsani A., Ostadrahimi A., Ghasempour Z. (2022). Atmospheric cold plasma effect on quality attributes of banana slices: Its potential use in blanching process. Innov. Food Sci. Emerg..

[71] Harrat R., Bourzama G., Sadrati N., Zerroug A., Burgaud G., Ouled-Haddar H., Soumati B. (2024). A comparative study on biodegradation of low density polyethylene bags by a *Rhizopus arrhizus* SLNEA1 strain in batch and continuous cultures. Braz. J. Microbiol..

[72] Zhao L. (2024). Research Progress on Physical Preservation Technology of Fresh-Cut Fruits and Vegetables. Horticulturae.

[73] Putnik P., Barba F.J., Lorenzo J.M., Gabrić D., Shpigelman A., Cravotto G. (2017). An Integrated Approach to Mandarin Processing: Food Safety and Nutritional Quality, Consumer Preference, and Nutrient Bioaccessibility. Compr. Rev. Food Sci. Food Saf..

[74] Soni A., Choi J., Brightwell G. (2021). Plasma-Activated Water (PAW) as a Disinfection Technology for Bacterial Inactivation with a Focus on Fruit and Vegetables. Foods.

